# Reentrant Resistive Behavior and Dimensional Crossover in Disordered Superconducting TiN Films

**DOI:** 10.1038/s41598-017-01753-w

**Published:** 2017-05-11

**Authors:** Svetlana V. Postolova, Alexey Yu. Mironov, Mikhail R. Baklanov, Valerii M. Vinokur, Tatyana I. Baturina

**Affiliations:** 1 A. V. Rzhanov Institute of Semiconductor Physics SB RAS, Novosibirsk, 630090 Russia; 2 Department of Physics, Novosibirsk State University, Novosibirsk, 630090 Russia; 3 North China University of Technology, Beijing, 100144 China; 4 Argonne National Laboratory, Materials Science Division, Lemont, IL 60439 USA; 5 Departamento de Fisica de la MateriaCondensada, Instituto de Ciencia de Materiales Nicolas Cabrera and Condensed Matter Physics Center (IFIMAC), Universidad Autonoma de Madrid, Madrid, E-28049 Spain

## Abstract

A reentrant temperature dependence of the normal state resistance often referred to as the *N*-shaped temperature dependence, is omnipresent in disordered superconductors – ranging from high-temperature cuprates to ultrathin superconducting films – that experience superconductor-to-insulator transition. Yet, despite the ubiquity of this phenomenon its origin still remains a subject of debate. Here we investigate strongly disordered superconducting TiN films and demonstrate universality of the reentrant behavior. We offer a quantitative description of the *N*-shaped resistance curve. We show that upon cooling down the resistance first decreases linearly with temperature and then passes through the minimum that marks the 3D–2D crossover in the system. In the 2D temperature range the resistance first grows with decreasing temperature due to quantum contributions and eventually drops to zero as the system falls into a superconducting state. Our findings demonstrate the prime importance of disorder in dimensional crossover effects.

## Introduction

**Figure 1:**
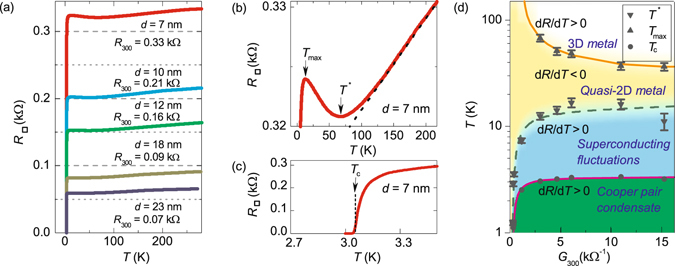
Reentrant temperature dependence of the resistance and phase diagram. (**a**) Resistance per square vs. temperature for five TiN films with different thicknesses *d* and different resistances at room temperature *R*_300_. (**b**,**c**) The enlarged areas from panel (a) for sample *d* = 7 nm. (**b**) Magnified representation of *R*(*T*) above the superconducting transition. All the samples exhibit the same behaviour. Arrows mark temperatures T* and *T*_*max*_. Dashed line corresponds to *R* ∝ *T*. (**c**) Magnified representation of *R*(*T*) near the superconducting transition. Arrow marks the superconducting critical temperature *T*_*c*_ determined from the quantum contribution fits (see Fig. 3a and details in the text). (**d**) The phase diagram for low-*T*_*c*_ thin TiN films in conductance (*G*_300_ = 1/*R*_300_)-temperature (*T*) coordinates. Temperatures T*, *T*_*max*_, *T*_*c*_ separate four distinct regimes of *R*(*T*). The five points at higher *G* correspond to films in (**a**). Three points for lower *G* (i.e. lower *T*_*c*_) corresponds to films from ref. 14, which have no T* value since in these films *R*(*T*) increases with cooling from room temperature.

Reentrant temperature dependence of the normal state resistance is found in a wide variety of disordered superconducting systems. With temperature decreasing, the resistance first decreases and then upturns upon further cooling down, and, finally, drops down to zero, see Fig. 1a,b. Similar *N*-shaped temperature dependence is observed in thin films of conventional superconductors PtSi^1^ and AlGe^2^. In the boron-doped granular diamond^3^ this behaviour is interpreted in the framework of an empirical model based on the metal-bosonic insulator-superconductor transitions induced by a granularity-correlated disorder. In high-T_*c*_ cuprates this behavior occurs often^4–10^, but is not thoroughly understood yet and is described in terms of the scaling functions^10, 11^ or attributed to the emergence of the pseudogap phase^12^. As cuprates are generically a stack of conducting CuO plains, a natural idea arises that it is the study of their elemental structural unit, two-dimensional disordered superconductor, that may provide a critical insight into the physics of high-*T*_*c*_.

Here we undertake a careful study of the strongly disordered ultrathin TiN superconducting film and find that its reentrant behavior occurs due to combined effects of the crossover from the 3D behavior determined by the Bloch-Grüneisen law to the quasi-2D behavior governed by competing quantum contributions to conductivity. We show that the shift from the downturn to upturn of the resistance occurs once the thermal coherence length *L*_*T*_ matches the thickness of the film *d*. We summarize our results by phase diagram in the conductance-temperature coordinates. Our findings demonstrate the important role of disorder in dimensional crossover effects.

## Experimental Techniques

The data are taken on thin, 7 ≤ *d* ≤ 23 nm, TiN films formed on a Si/SiO_2_ substrate by the atomic layer deposition. The films are atomically smooth polycrystalline with the densely-packed crystallites and are similar to those analyzed in refs 13, 14. Here we deal with homogeneously disordered films i.e. films with ${k}_{F}l\mathop{ < }\limits_{ \tilde {}}10$ and characteristic size of structural inhomogeneities *δx* less than characteristic lengths of the system. In this case the most reliable measure of disorder is the resistance per square ${R}_{\square }$ (in other words the conductance *G*) of the system^15^. The parameters of the sample are calculated in the approximation of the parabolic dispersion law, from the superconducting critical temperature *T*_*c*_, and carrier density *n* is found from the measurements of the Hall effect at *T* = 10 K. The samples are patterned into bridges 50 *μ*m wide and 250 *μ*m long. Transport measurements are carried out using the low-frequency ac technique in a four-probe configuration.

## Experimental Results

Temperature dependencies of the resistance per square, ${R}_{\square }(T)$, at zero magnetic field for TiN films with different thicknesses and different resistances at room temperature are shown in Fig. 1a. As a function of the decreasing temperature, the resistance first decreases (*dR*/*dT* > 0) linearly at high temperatures (see Fig. 1b), then deviates upwards from *R* ∝ *T* reaching the minimum at some temperature T*. The temperature T* grows with the film resistance *R*_300_, i.e. with the increasing degree of disorder (Fig. 1d). Upon further cooling, ${R}_{\square }(T)$ increases (*dR*/*dT* < 0) and passes the local maximum at *T*_*max*_ to drop (*dR*/*dT* > 0) down to zero resistance *below* the superconducting critical temperature *T*_*c*_ (see Fig. 1c). The superconducting transition temperature *T*_*c*_ is defined as an adjusting parameter in quantum contributions to conductivity^14^, see also the discussion below, and is located at the foot of the ${R}_{\square }(T)$ curve. A crude estimate gives $R({T}_{c})\simeq 0.1{R}_{max}$, where *R*_*max*_ is the resistance at *T*_*max*_^14^. The temperature *T*_*c*_ decreases with the decreasing films thickness, i.e. with the growth of the films’ resistance per square at room temperature *R*_300_. The latter is a convenient parameter to characterize the degree of disorder in films. Figure 1d summarizes our observations into a phase diagram in the conductance-temperature coordinates (*G*; *T*) where *G* = 1/*R*_300_. The obtained phase diagram of TiN films resembles those for the high-T_*c*_ superconductors^16, 17^ with the doping *x* being replaced by conductivity, *G*. The diagram comprises the four regions corresponding to the distinct regimes of *R*(*T*) dependence. These regions are separated by the characteristic temperatures T*, *T*_*max*_, and *T*_*c*_.

## Discussion

**Figure 2:**
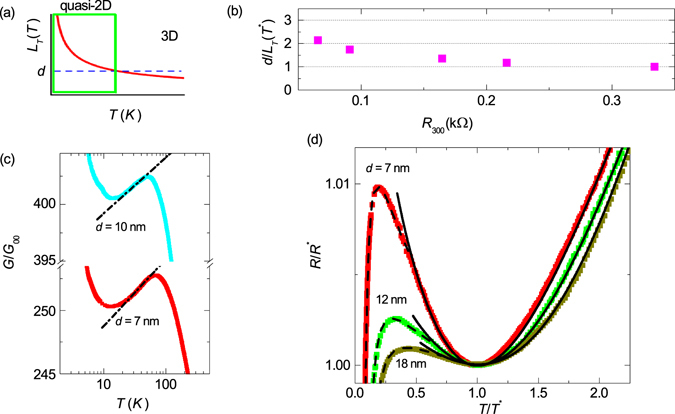
Dimensional crossover and quantum contributions fits. (**a**) A sketch of the temperature dependence of thermal coherence length ${L}_{T}\propto 1/\sqrt{T}$ (solid line). The rectangle confines the region where *L*_*T*_ exceeds the film thickness *d* (dashed line). (**b**) The ratio of the film thickness to thermal coherence length *d*/*L*_*T*_(T*) at the crossover temperature T* *vs*. resistance at room temperature *R*_300_. (**c**) The dependence *R*(*T*) from Fig. 1a replotted as a dimensionless conductance *G*/*G*_00_ as function of the temperature in the logarithmic scale for samples *d* = 10 nm and *d* = 7 nm. Dash-dotted lines correspond to *G*/*G*_00_ ∝ ln *T*. (**d**) The reduced resistance *R*/R* vs reduced temperature *T*/T*, where T* and R* are the temperature and resistance at the local minimum. Symbols stand for experimental data, solid lines are fits by Eqs (7) and (8). Note, that three samples with lowest *T*_*c*_ did not show a R(T) minimum. Dashed lines are fits accounting for all the quantum contributions to conductivity (see Fig. 3a and the discussion in the text).

The linear high-temperature behavior of *R*(*T*) in Fig. 1a,b follows from the high-temperature asymptote of the so-called Bloch-Grüneisen formula^18–21^

1 $${R}_{{\rm{B}}{\rm{G}}}(T)=C\cdot {(\frac{T}{{\rm{\Theta }}})}^{5}{\int }_{0}^{{\rm{\Theta }}/T}\frac{{z}^{5}dz}{({e}^{z}-1)(1-{e}^{-z})},$$

where Θ is the Debye temperature and *C* is the material constant (see Methods for details). The approximation *R*_*BG*_ ∝ *T* holds down^18^ to temperatures $T\simeq {\rm{\Theta }}/3$. With the decreasing temperature, the upturn from the foregoing linear dependence occurs and upon passing the minimum at T* the resistance starts to increase. This growth of the resistance resembles the quasi-2D metallic behaviour dominated by the quantum contributions to conductivity. One thus can justly conjecture that T* marks the crossover from the 3D to the quasi-2D behavior of *R*(*T*) around T*. To check it let us compare the film thickness *d* and two characteristic lengths of the system: (i) the phase-coherence length,

2 $${L}_{\phi }=\sqrt{D{\tau }_{\phi }},$$

where *τ*_*ϕ*_ is the phase decoherence time; (ii) the thermal coherence length,

3 $${L}_{T}=\sqrt{2\pi \hslash D/({k}_{{\rm{B}}}T)},$$

where *k*_B_ is the Boltzmann constant, and diffusion coefficient *D* is refs 22, 23:

4 $$D=(\pi /2\gamma )({k}_{{\rm{B}}}{T}_{c}/e{B}_{c2}(0)),$$

where *γ* is Euler’s constant *γ* = 1.781, and *B*_*c*2_(0) is the upper critical field at *T* = 0 (see SI for details). The quasiparticle description holds for ${k}_{{\rm{B}}}T\gg \hslash /{\tau }_{\phi }$ ^24^ which implies ${L}_{T}\ll {L}_{\phi }$. Therefore, it is the thermal coherence length *L*_*T*_ that controls the effective dimensionality of the system (Fig. 2a). When the length *L*_*T*_ exceeds the thickness *d* (provided that the lateral size $L\gg d$) the system becomes two-dimentional with respect to effects of the electron-electron interaction^25^. The ratio *d*/*L*_*T*_(T*) as a function of the sample resistance at room temperature *R*_300_ is shown in Fig. 2b. The temperature *T* = T* marks the moment where becomes $d\simeq {L}_{T}$ hence the 3D–2D crossover, so that $d\mathop{ > }\limits_{ \tilde {}}{L}_{T}$ at *T* > T* and the system is three dimensional, whereas at *T* < T*, $d\mathop{ < }\limits_{ \tilde {}}{L}_{T}$ and the system turns two-dimensional.

The same data as in Fig. 1b but replotted as the dimensionless conductance *G*/*G*_00_ are shown in Fig. 2c in the semilogarithmic scale. This representation reveals the logarithmic temperature dependence of the conductance which is typical for two-dimensional (2D) metal where the effects of weak localization (WL) and electron-electron interaction in diffusion channel (ID) are enhanced by dimensionality^14, 25^. In the 2D case the WL and ID contributions to conductivity are

5 $${\rm{\Delta }}{G}^{WL+ID}={\rm{\Delta }}{G}^{WL}+{\rm{\Delta }}{G}^{ID}={G}_{00}A\,\mathrm{ln}\,({k}_{B}T\tau /\hslash ),$$

6 $$A=ap+{A}_{ID},$$

where ${G}_{00}={e}^{2}/\mathrm{(2}{\pi }^{2}\hslash )$, *a* = 1 provided the spin-orbit scattering is neglected (${\tau }_{\phi }\ll {\tau }_{so}$) and *a* = −1/2 for ${\tau }_{\phi }\gg {\tau }_{so}$, *p* is the exponent in the temperature dependence of the phase decoherence time ${\tau }_{\phi }\propto {T}^{-p}$, and ${A}_{ID}\simeq 1$ is a constant accounting for the Coulomb screening. At low temperatures where electron-electron scattering dominates, ${\tau }_{\phi }\propto {T}^{-1}$, i.e. *p* = 1. At high temperatures where the electron-phonon interaction becomes relevant, *p* = 2 in the presence of disorder. Hence at high temperatures $A\mathop{ < }\limits_{ \tilde {}}3$ is expected for homogeneously disordered films.

We are now equipped to fully describe the behavior of *R*(*T*) above *T*_*max*_ as a result of superposition of quantum contributions to conductivity (WL+ID) and the Bloch-Grüneisen law. In the linear regime they just add to each other:

7 $$R(T)={R}_{{\rm{BG}}}(T)+{R}_{res},$$

where *R*_BG_(*T*) is defined in Eq. (1), *R*_*res*_ is a residual resistance. In our case this residual resistance is given by

8 $${R}_{res}=1/({R}_{0}^{-1}+{\rm{\Delta }}{G}^{WL+ID}),$$

where Δ*G* ^*WL*+*ID*^ is defined in Eq. (5) and *R*_0_ is the residual resistance due to defect scattering, proportional to normal resistance of the sample (see Methods). Figure 2d demonstrates an excellent fitting by Eq. (7) to the experimental data, capturing the original decrease of *R*(*T*) with temperature decreasing, the minimum, and the subsequent growth. The parameter *A* in Eq. (5) is *A* = 2.85 ± 0.15, which agrees with theoretical predictions. The Debye temperature is Θ = 450 ± 10 K i. e. is 30% less than Θ in the bulk material^26^ (the decrease of the Debye temperature with the decrease of the system dimensionality has been reported before^27–29^). The values of *C* are of same orders as those previously found in other superconducting materials^30, 31^. To conclude here, the minimum in *R*(*T*) results from the dimensional crossover between the 3D behavior governed by the Bloch-Grüneisen formula and the 2D region, where *R*(*T*) is controlled by quantum contributions. The fit works perfectly down to temperatures $T\mathop{ < }\limits_{ \tilde {}}10{T}_{c}$ where superconducting fluctuations (Δ*G* ^*SF*^) start to dominate.

**Figure 3:**
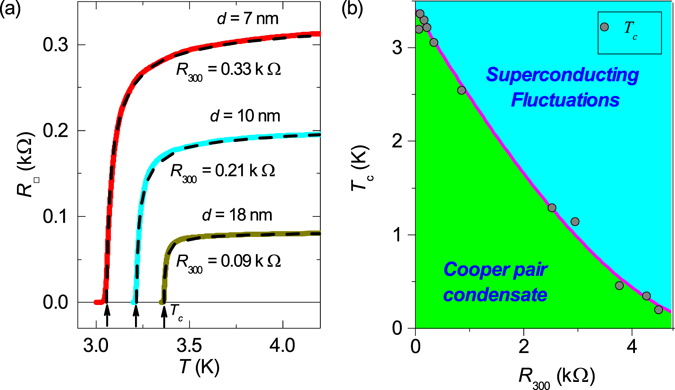
Superconducting critical temperature. (**a**) Determination of *T*_*c*_ from quantum contributions to the conductivity. Solid lines: experimental resistances per square vs. temperature for three TiN film with different thicknesses *d* and different resistances at room temperature *R*_300_. Dashed lines are the same as in Fig. 2d, the fits account for all quantum contributions to conductivity. Arrows mark the respective superconducting critical temperatures *T*_*c*_. (**b**) The superconducting critical temperature *T*_*c*_ *vs*. *R*_300_ for the TiN films shown in Fig. 1a and published in refs 14, 46, 47. The solid line is the theoretical fitting by Eq. (9) with the adjustable parameter $\gamma =\,\mathrm{ln}\,[\hslash /(k{T}_{c0}\tau )]=5.73$, where *T*_*c*0_ = 3.4 K and *τ* = 7.3 · 10^−15^ s.

Below T* the resistance increases, reaches the maximum, and, finally, decreases down to zero (Figs 2d and 3a). This non-monotonic *R*(*T*) is similar to that of thinner high-resistance samples and has been fully analyzed^14^. Quantum correction formulas fit perfectly the experimental data. In passing, following^14^, these fits, in which *T*_*c*_ plays the role of the adjustable parameter, yield the precise value of the critical temperature (Fig. 3a). Figure 3b shows that the suppression of the critical temperature *T*_*c*_ with the increase of the normal state resistance *R*_300_ follows the celebrated Finkelstein’s formula^32^:

9 $${\rm{l}}{\rm{n}}(\frac{{T}_{c}}{{T}_{c0}})=\gamma +\frac{1}{\sqrt{2r}}{\rm{l}}{\rm{n}}(\frac{1/\gamma +r/4-\sqrt{r/2}}{1/\gamma +r/4+\sqrt{r/2}}),$$

where *r* = *G*_00_ · *R*_*sq*_, *R*_*sq*_ is the resistance per square, $\gamma =\,\mathrm{ln}\,[\hslash /(k{T}_{c0}\tau )]$. The best fit of Eq. (9) to experimental data is achieved at *T*_*c*0_ = 3.4 K, *τ* = 7.5 · 10^−15^ s, yielding *γ* = 5.73, the found values agreeing fairly well (see Fig. 3b) with the earlier data^21^ for TiN.

Our results are summarized in a phase diagram (Fig. 1d) displaying the effective dimensionality of the system and the corresponding mechanisms of the conductivity. We identify four distinct regimes. At high temperatures, *T* > T*, where $d\mathop{ > }\limits_{ \tilde {}}{L}_{T}$, the resistance depends linearly on temperature and the system is a 3D metal. The boundary for this region is calculated from the condition *dR*/*dT* = 0, see Eqs (1) and (7). Down in temperature, at *T* < T*, $d\mathop{ < }\limits_{ \tilde {}}{L}_{T}$, and the system becomes two-dimensional. The resistance in this region is dominated by quantum contributions from electron-electron interaction and weak localization. The boundary *T*_*max*_(1/*R*) separating this region and the region controlled by superconducting fluctuations is found from the condition ${\rm{\Delta }}{G}^{SF}\simeq {\rm{\Delta }}{G}^{WL+ID}$ as in ref. 14 (see SI). Below *T*_*max*_ electronic transport in the system is governed by superconducting fluctuations. Finally, the line that marks formation of the Cooper condensate is calculated from Eq. (9).

Let us discuss the high-*G* region of this diagram. The temperature dependence *R*(*T*) of films with higher *G*_300_ shows wider and less-pronounced minimum at T* and maximum *T*_*max*_, that is reflected by increase of error bars with *G*_300_. The experimental *T*_*max*_(*G*) dependence seems to show a maximum and subsequent decease. This is because of the point *T*_*max*_ coming from the last sample with *d* = 23 nm. This untoward sample segregates from the common trend in saturation *T*_*max*_(*G*). However, T*(*G*) corresponding to this sample obeys the general saturation trend and demonstrates the reasonable *d*/*L*(T*) value in Fig. 2b supporting the idea of 3D–2D dimensional crossover taking place in minimum in *R*(*T*). The theoretical dependence *T*_*max*_(*G*) saturates with increasing *G* (dashed line in Fig. 1d) as well as theoretical T*(*G*). So the natural question arises: what would we observe at higher *G*, that we did not test in experiment? It is clearly seen that for both quantities T* and *T*_*max*_, the error of determination increases with *G*. So, it seems like at higher *G* both this features of *R*(*T*) would be blurred and the dimensional crossover would gradually fade away towards thicker films that would remain three-dimensional at all temperatures. On the other hand, even in 3D superconducting films, there will be the *N*-shaped region of *R*(*T*), weakly pronounced though, associated with saturation of Drude conductivity and the influence of 3D quantum contributions to conductivity. Unfortunately we do not have films with higher conductance *G* to make decisive measurements.

Now we briefly compare the behaviour of TiN films and cuprates. The similarity of cuprates and thin films of conventional superconductors has been demonstrated in a various experiments. Namely, ultrathin disordered films of TiN exhibit the pseudogap pretty similar to that of the high-T_*c*_^33^. The former stems from the appreciable depletion of the density of the electronic states (DOS) by superconducting fluctuations favored by two-dimensionality and disorder. The behavior of the mid-infrared optical conductivity of TiN is similar to pseudogap features of high-T_*c*_ and is described in terms of fluctuation dominated 2D transport^34^. Several studies pointed a peak in the Nernst effect in both high-*T*_*c*_^35^ and thin-films of conventional superconductors^36, 37^. The *N*-shaped temperature dependence offers another example of similarity between the high-T_*c*_ and disordered thin films. However, the situation in high-*T*_*c*_ is by far more complicated because of other interfering orders, including various forms of charge-density-waves, spin-density-waves, and electron-nematic order^38^, to name a few. For example, in underdoped cuprates YBCO the *N*-shaped temperature dependences of *R*(*T*) is attributed to formation of 1D conducting charge stripes, and the experimental data seem to well agree with the expected scaling behavior^11^. In BSCCO-2212:La above the critical doping, the reentrant behavior is well described by the model of the two-component scaling function that describes the coexistence of the weakly insulating phase and the superconductive fluctuating phase consistent with the electronic phase-separation mechanism driven by carrier-carrier correlations^10^. This scaling was not observed in our data (see SI Fig. 3), despite that one would expect TiN films to demonstrate phase separation on the brink of superconductor-insulator transition as well^39^. Another complication in comparing normal state properties in cuprates and those in superconducting thin films may stem from the supposed non-Fermi-liquid nature of electrons in the former. This means that the analysis of the *N*-shaped *R*(*T*) based on the standard electron-phonon scattering (Eq. (1)) and quantum corrections (Eq. (5)) considerations should be applied to cuprates with certain reservations and caution. Yet, the observation of 2D-like weak localization was reported, for example, in nonsuperconducting overdoped TlBCO single crystals^40^. Since in high-*T*_*c*_ the conductivity along the layers is much greater than that across the layers, the electrons there can be viewed as confined to individual layers^41^. Therefore, the corresponding quantum contributions are described by the same Eqs (5) and (6) butwith $A\gg 1$ ^42^. As for the fluctuation phenomena, the superconducting fluctuations above *T*_*c*_ are prominently present in high-*T*_*c*_ compounds because of their extreme anisotropy^43, 44^. Concluding here the hole situation in cuprates calls for more thorough research.

## Conclusion

We construct the phase diagram displaying the effective dimensionality of the TiN films and the corresponding mechanisms of the conductivity. We show that the minimum in *R*(*T*) marks the crossover between the 3D and 2D behaviours at the temperature where the thermal coherence length *L*_*T*_ compares to the film thickness. We demonstrate that the total *N*-shaped temperature dependence of the TiN film resistance results from the intertwined effects of the Bloch-Grüneisen law and quantum contributions to conductivity. The observed *N*-shaped dependence resembles strikingly the temperature behaviour of the resistance found in a wide variety of disordered systems and materials.

## Methods

### Numerical parameters

The material constant *C* in Eq. (1) is ref. 20:

10 $$C=\frac{m}{{e}^{2}n}\cdot \frac{1}{d}\cdot \frac{3\pi {c}_{0}^{2}m}{\hslash {k}_{B}{\rm{\Theta }}M{a}^{3}n},$$

here *m* and *e* are mass and charge of an electron respectively, *n* is electron density, *M* is mass of an atom, *a* is lattice constant, *c*_0_ is the material parameter depending on the velocity of electrons at the Fermi surface; usually *c*_0_ = 1 ÷ 10 eV^20^. Taking *m* = 2*m*_0_^45^, the average TiN atomic mass $M=(1/2)({M}_{{\rm{Ti}}}+{M}_{{\rm{N}}})\simeq 5\cdot {10}^{-26}\,{\rm{kg}}$, lattice constant $a\simeq 0.4\,{\rm{nm}}$ and ${c}_{0}\simeq 8\,{\rm{eV}}$ the estimated values of *C* match those determined from fitting of experimental *R*(*T*) dependencies.

**Table:** 

*d*, nm	*R*_300_, Ω	*k*_*F*_ *l*	*D*, cm^2^/s	*C*, Ω	*R*_0_, Ω
7	334	4.9	0.68	163.5	314.5
10	216	5.8	0.76	159	198.7
12	165	6.2	0.8	105	150.9
18	90	7.4	0.82	95	81.5
23	65	7.9	0.87	69	58.8

## Electronic supplementary material

Supplementary Information: \\ Reentrant Resistive Behavior and Dimensional Crossover in Disordered Superconducting TiN Films
