# Supplementary material for: Reentrant Resistive Behavior and Dimensional Crossover in Disordered Superconducting TiN Films
Source: Sci Rep. 2017 May 11;7:1718. doi: 10.1038/s41598-017-01753-w (PMC5431868; doi:10.1038/s41598-017-01753-w)
Supplement: Supplementary file 1 — Supplementary Information: \\ Reentrant Resistive Behavior and Dimensional Crossover in Disordered Superconducting TiN Films [file 41598_2017_1753_MOESM1_ESM.pdf]

Supplementary Information:

Reentrant Resistive Behavior and Dimensional Crossover in Disordered Superconducting TiN Films

S. V. Postolova,[1,2] A. Yu. Mironov,[1,2] M. R. Baklanov,[3] V. M. Vinokur,[4] and T. I. Baturina[1,2]

[1]*A. V. Rzhanov Institute of Semiconductor Physics SB RAS, 13 Lavrentjev Avenue, Novosibirsk, 630090 Russia*
[2]*Novosibirsk State University, Pirogova str. 2, Novosibirsk 630090, Russia*
[3]*North China University of Technology, Beijing, 100144, China*
[4]*Materials Science Division, Argonne National Laboratory, Argonne, Illinois 60439, USA*

The crystallites size distribution of films follows the lognormal distribution

$$f(x) \propto \frac{1}{x\sigma\sqrt{2\pi}} \cdot e^{-\frac{(\ln x-\mu)^2}{2\sigma^2}}, \qquad (1)$$

where values of μ, σ and mode $x_M = \exp(\mu - \sigma^2)$, the value that appears most often in a set of data, are listed in table.

Parameters of crystallites size distribution:

d, nm	3.6	5	18
x_M, nm	4.5 ± 0.2	3.3 ± 0.2	3.7 ± 0.2
μ	1.6 ± 0.03	1.3 ± 0.03	1.4 ± 0.03
σ	0.26 ± 0.03	0.32 ± 0.03	0.35 ± 0.03

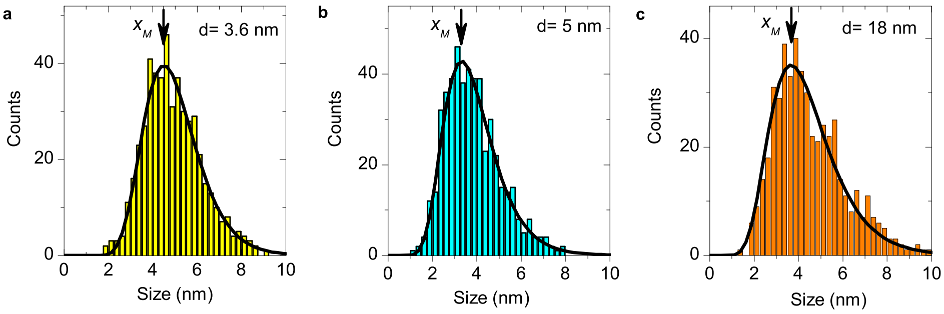

Figure S 1: Grain size distribution. Crystallites size distribution of the films d=3.6 nm **(a)**, d=5 nm **(b)**, d=18 nm **(c)**.

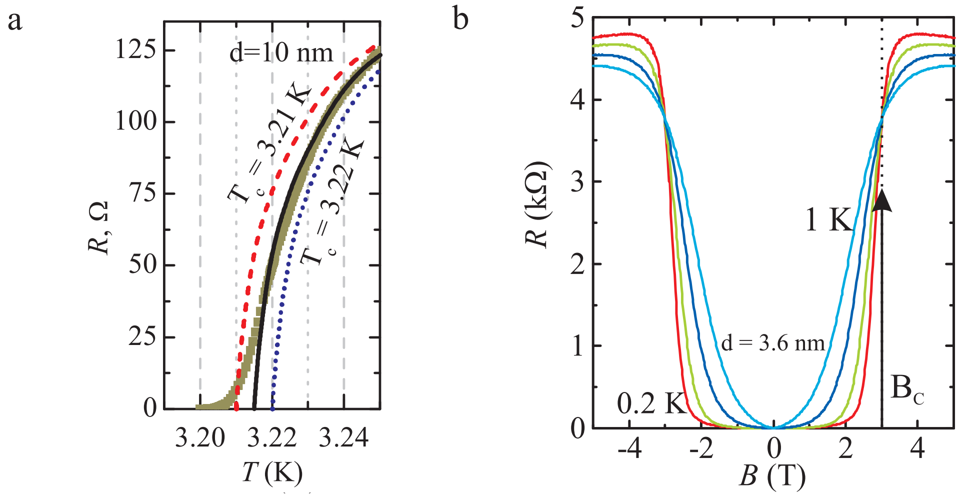

Figure S 2: **Determination of T_c and $B_{c2}(0)$.** (a) Resistance per square vs. temperature for $d = 10$ nm TiN film. Symbols: experimental dependence. Solid line — fit accounting for all the quantum contributions to conductivity with adjusting parameter $T_c = 3.215$ K; dashed line — $T_c = 3.21$ K; dotted line — $T_c = 3.22$ K. (b) Resistance vs. magnetic field. From the crossing point we extract B_c. As it was shown in work [1] $B_{c2}(0) \simeq B_c/1.04$.

We calculate the diffusion coefficient $D = (\pi/2\gamma)(k_{\mathrm{B}}T_c/eB_{c2}(0))$, where T_c is the superconducting critical temperature, which is defined as an adjusting parameter in fits of $R(T)$ quantum contributions (Fig. 3 (a)), $B_{c2}(0)$ — upper critical magnetic field, determined from $R(B)$ dependencies (Fig. 3 (b)).

Calculation of $T_{max}(G)$ dependence

The starting point of this calculation is expression for resistance of the disordered system which is the sum of all quantum contributions added to the bare Drude conductivity $G_\square$:

$$R(T) = [G_\square + \Delta G^{WL+ID}(A,T) + \Delta G^{SF}(T_c,\delta)]^{-1}, \tag{2}$$

where $\Delta G^{WL+ID} < 0$ and superconducting fluctuations $\Delta G^{SF} > 0$. One sees that although the **SF** contributions alone would have resulted in the monotonic behaviour of the resistance (with dR/dT¿0), the contributions from **WL+ID** processes make $R(T)$ become non-monotonic and exhibit a maximum at the some temperature, T_{max}, of about of few T_c (see Fig.2d in main text). The correction from superconducting fluctuations depends on pair-breaking parameter δ which in turn depends on temperature T and phase coherence time τ_φ

$$\delta = \frac{\pi\hbar}{8kT\tau_\varphi}. \tag{3}$$

At low temperatures where electron-electron scattering dominates,

$$\tau_\varphi^{-1} = \frac{\pi kT}{\hbar}\frac{e^2R}{2\pi^2\hbar}\ln\frac{e^2R}{\pi\hbar} \tag{4}$$

and δ depends only on $R_\square$

$$\delta = \frac{e^2R_\square}{16\hbar}\ln\frac{\pi\hbar}{e^2R_\square}. \tag{5}$$

We use $G_\square = 1/R_\square$ as an input parameter. We calculate δ, calculate corresponding T_c from Finkelstein formula (see main text), take $A = 2.85$ for ΔG^{WL+ID} (according to experimental result discussed in main text), calculate theoretical $G(T)$, and extract the T_{max} value. Reproducing this procedure for different value of input parameter $G_\square$ we get the $T_{max}(G)$ dependence.

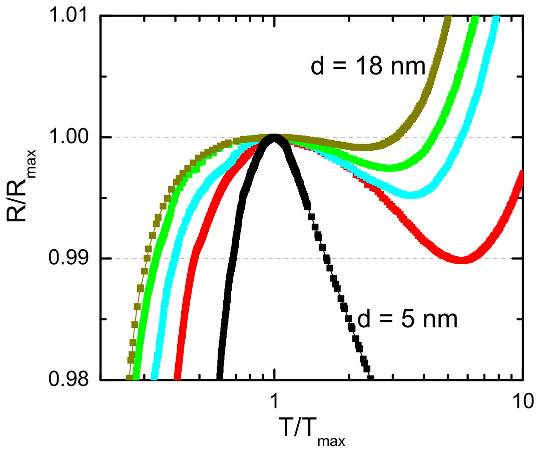

Figure S 3: The failed scaling of superconducting curves analogous to scaling presented in work [2].

SUPPLEMENTARY REFERENCES

[1] Gantmakher, V. F., Ermolov, S. N., Tsydynzhapov, G. E., Zhukov, A. A. & Baturina, T. I. Suppression of 2D superconductivity by the magnetic field: quantum correction vs superconductor-insulator transition. *JETP Letters* **77**, 424 (2003).

[2] Oh, S., Crane, T. A., Van Harlingen, D. J. & Eckstein, J. N. Doping Controlled Superconductor-Insulator Transition in $Bi_2Sr_{2-x}La_xCaCu_2O_{8+\delta}$. *Phys. Rev. Lett.* **96**, 107003 (2006).
